# Use of cellular immunotherapy for treatment of refractory acute myeloid leukemia

**DOI:** 10.1186/2051-1426-1-S1-P7

**Published:** 2013-11-07

**Authors:** Loren Fast, John Reagan, Peter Quesenberry

**Affiliations:** 1 Medicine, Rhode Island Hospital, Providence, RI, USA

## 

A previous clinical trial carried out by our group in which high numbers of haploidentical donor lymphoid cells containing CD3+ cells (1 - 2 x 10^8^ CD3+ cells/kg) were infused into recipients with refractory hematological malignancy found that 14/27 patients gave a response, 9 or which were major. These recipients rapidly developed a cytokine storm which was resolved, if needed, by administrating steroids. No persisting donor cells could be detected at 2 weeks following infusion. After gaining FDA and IRB approval, this cellular immunotherapy trial was restarted. One of the goals of the trial was to define the effector mechanisms that were playing a role in anti-leukemic responses. The results from the first recipient enrolled indicated that right after the infusion, 11.3% of peripheral blood mononuclear cells were donor cells as defined by donor-specific anti-HLA antibodies. Donor cell levels had decreased to 0.95% on day 7. Increased expression of granzymes and perforin by both donor and recipient cells especially 2 - 3 days after infusion indicated that these cells exhibited increased cytolytic activity. In addition, both donor and recipient CD4+ cells were found to express increased levels of CD134, and a large fraction of CD8+ cells expressed CD279 (PD-1). The role of cytolytic cells will be assessed further as additional patients are enrolled and the expression of activating or inhibitory receptors will be confirmed. These results could suggest additional therapeutic approaches to be tested for enhancing the anti-leukemic responses following cellular immunotherapy.

